# Supplementary material for: Classical center-surround receptive fields facilitate novel object detection in retinal bipolar cells
Source: Nat Commun. 2022 Sep 26;13:5575. doi: 10.1038/s41467-022-32761-8 (PMC9512824; doi:10.1038/s41467-022-32761-8)
Supplement: Supplementary file 1 — Supplementary Information [file 41467_2022_32761_MOESM1_ESM.pdf]

**Supplementary figures**

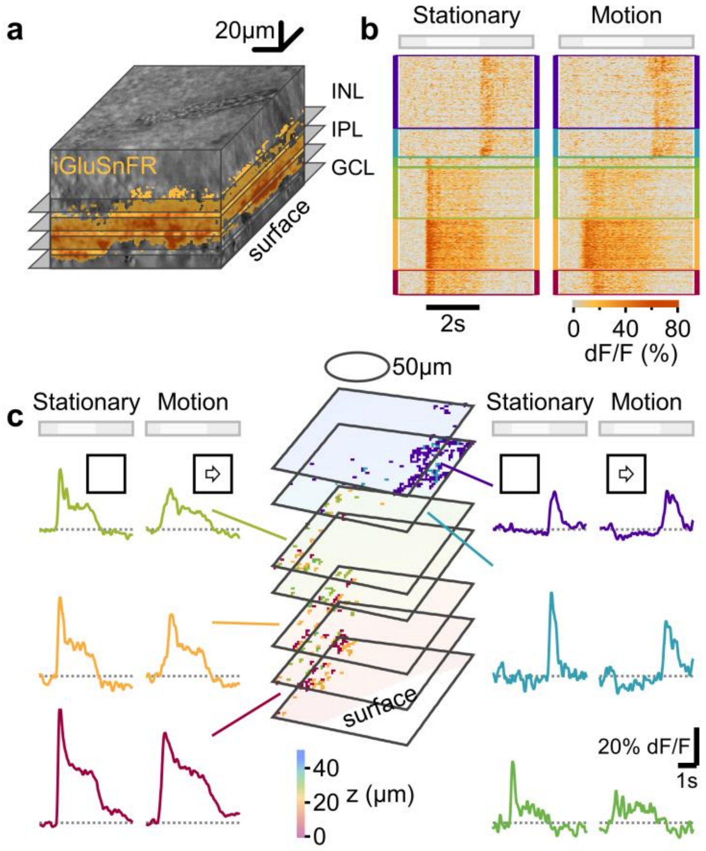


**Supplementary figure 1. Recording and analysis of light-driven glutamatergic responses in the inner retina. a** 3D reconstruction of a two-photon imaged retina, showing iGluSnFR expression in the inner retina (orange) and a schematic of the scan planes. INL, inner nuclear layer; IPL, inner plexiform layer; GCL, ganglion cell layer. **b** A heatmap illustrating the temporal responses to full-field stationary (left) and moving (right) bars recorded from representative pixels in the retina shown in **a**. Responses were clustered into ROIs based on the similarity of response waveforms. **c** The mean signals from the ROIs outlined in **b**. The middle panel shows the location of the pixels belonging to the ROI across scan planes. Ellipse-the approximate extent of a center RF region in BCs. Color-coding based on the mean IPL depth.

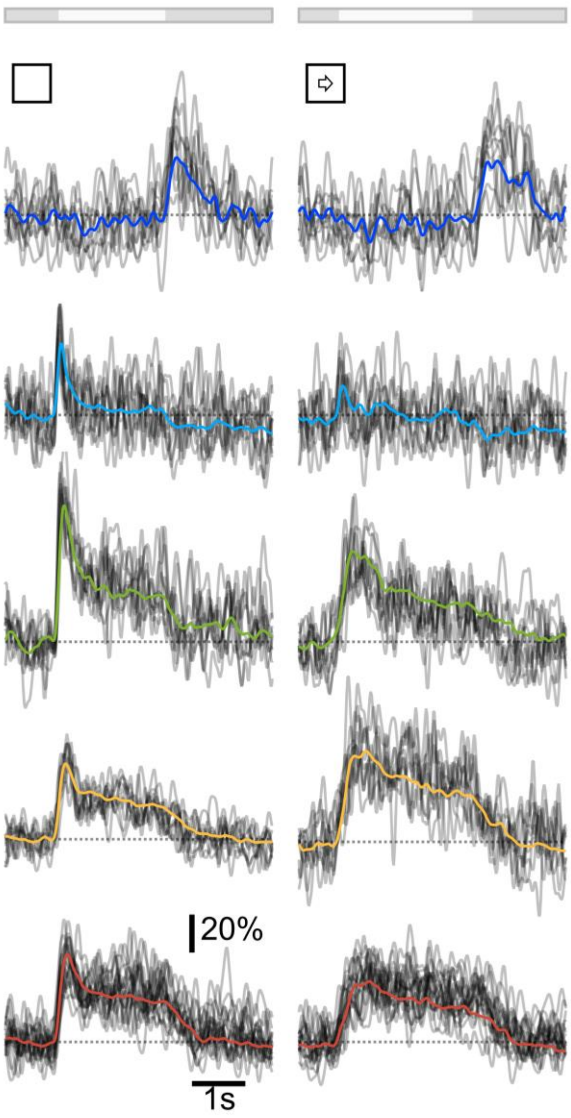


**Supplementary figure 2. Grouping of iGluSnFR signals.** Grey, ten representative traces of fluorescence change over time, computed in individual pixels belonging to 5 different iGluSnFR ROIs. The clustering algorithm compared the similarity between responses from individual pixels in the image that passed the inclusion criteria (methods).

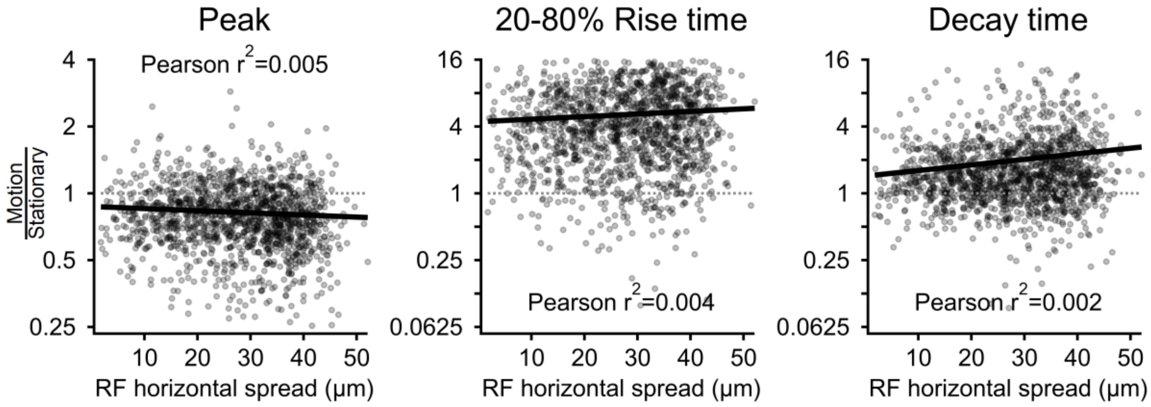


**Supplementary figure 3. Little effect of the spatial pixels spread on the extrapolated ROI dynamics.** The ratio between the peak (left), rise time (middle) and decay time (right) responses to full-field motion vs. stationary stimulation as a function of the SD of the horizontal pixel spread for all ROIs in the dataset. Solid lines, linear fits to the data.

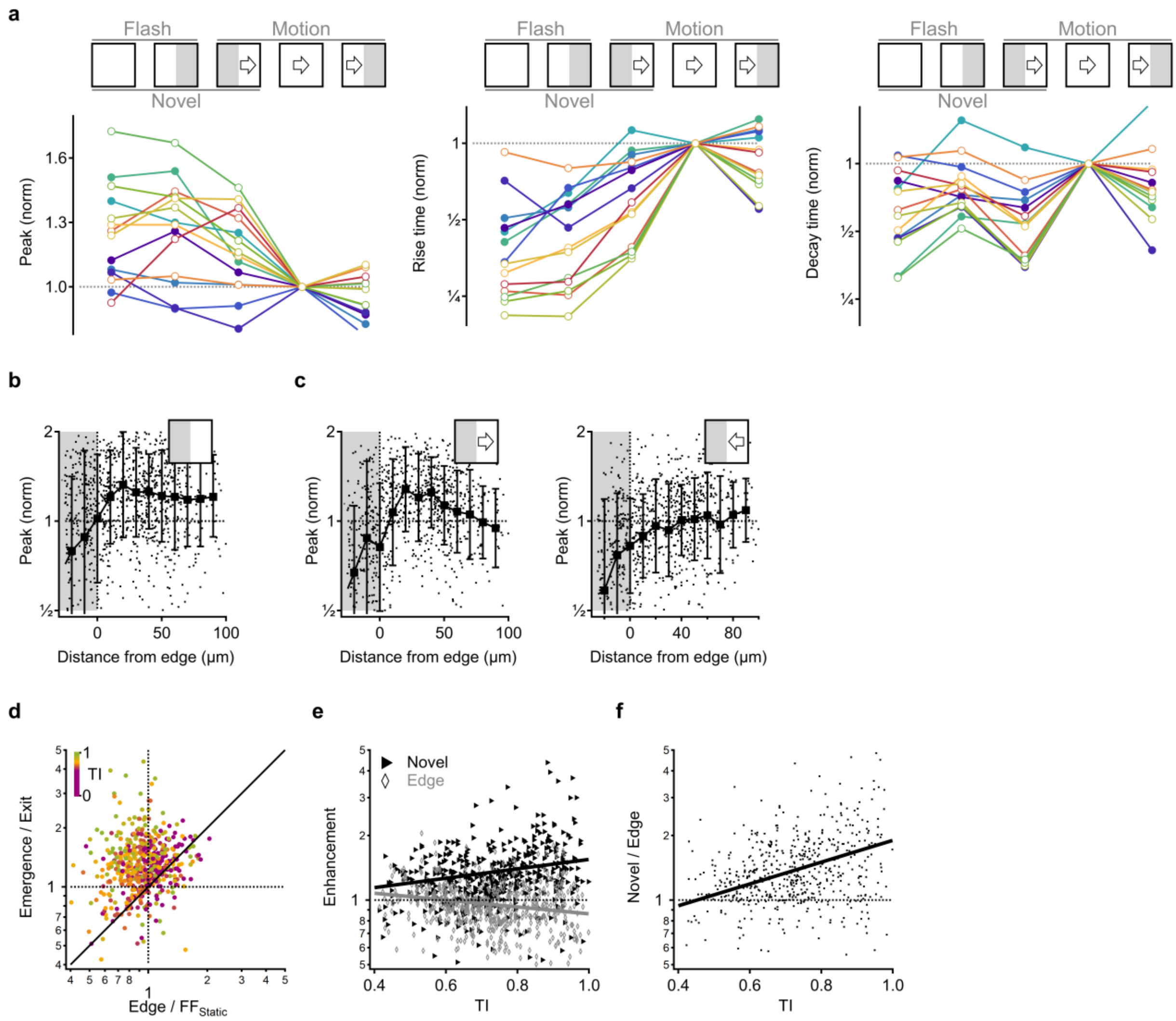


**Supplementary figure 4. Dynamics of glutamate responses near mask-stimulus boundary. a T**he mean peak amplitude (left), rise time (center), and decay time (right) of the fluorescent signals recorded in each of the glutamate clusters for the five visual conditions (full-field static flash, flash in the presence of a mask, motion from a mask, full-field motion, motion towards the mask), normalized by the corresponding attributes of the responses to full-field motion. Color coding as in **Fig. 1**. ON/OFF clusters are marked with open/filled circles, respectively. **b-c** The spatial extent of the edge effects for flashes (**b**) and moving stimuli (**c**). Dots mark the normalized (by full-field motion) response amplitude from all recorded ROIs as a function of their RF distance from the mask. Negative distances indicate RF position within the masked region (shaded). Solid line, the mean(±SD) normalized responses in 10 µm bins. Sample size as

in **Fig. 3**. **d-f** Emerging object detection is more prominent compared to stationary edge enhancement. **d** The relationship between novel object enhancement (measured here as the ratio between the peak of the ROI signal to emerging object vs. exiting objects) and edge enhancement (measured here as the ratio between the peak amplitude of the stationary response near mask-stimulus boundary vs. the response to the full-field flash). Color coding is by the transiency index calculated from the full-field static signals. **e** Novel object (black) and edge (grey) enhancement as a function of ROI transiency. **f** The ratio between novel object and edge enhancement vs. the transiency index. Solid lines in **e** and **f** indicate linear fits. More pronounced emerging motion enhancement was seen in transient ROIs. The majority of ROIs were tuned to the detection of novel objects as compared to the presence of static edges. Sample size for (**d-f**) as in **Fig. 1**.

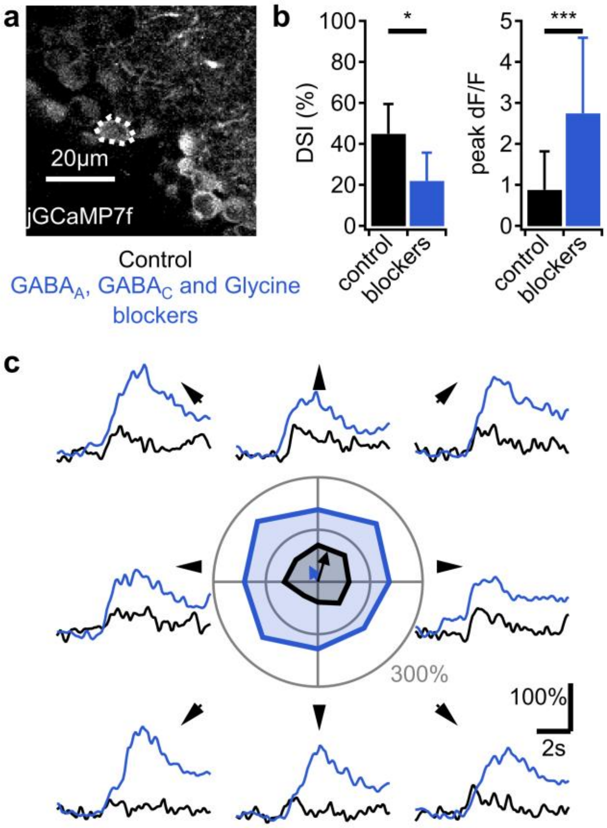


**Supplementary figure 5. Validation of inhibitory blockers effectiveness on responses of ganglion cells. a** jGCAMP7f was expressed in the ganglion cell layer of WT mice and ganglion cell activity was monitored in ex-vivo retina during stimulation with bright bars moving in 8 directions separated by 45˚. Direction selective cells were identified by having a DSI>10%. Following control recording in Ames solution, blockers of GABA$_A$, GABA$_C$ and glycine were added to the bath. **b** Comparison between the DSI values of DS ganglion cells (left, n=6) and peak fluorescence in all recorded ganglion cells (right, n=22 ROIs, 3 animals, 1 female, ages p49-78) in control solution (black) and after addition of inhibitory blockers (grey). *p=0.015,***p=$2\times10^{-4}$; paired t-test. Error bars-SD. **c** Example light responses from a DS ganglion cell outlined in **a**. The directional vector was computed as the vector sum of the peak responses to different directions. Color coding as in **b**. As expected from the role of inhibition in governing the size of DS in ganglion cells and in dampening neuronal responses in general, we observed reduced DS and larger calcium transients following the application of the drugs.

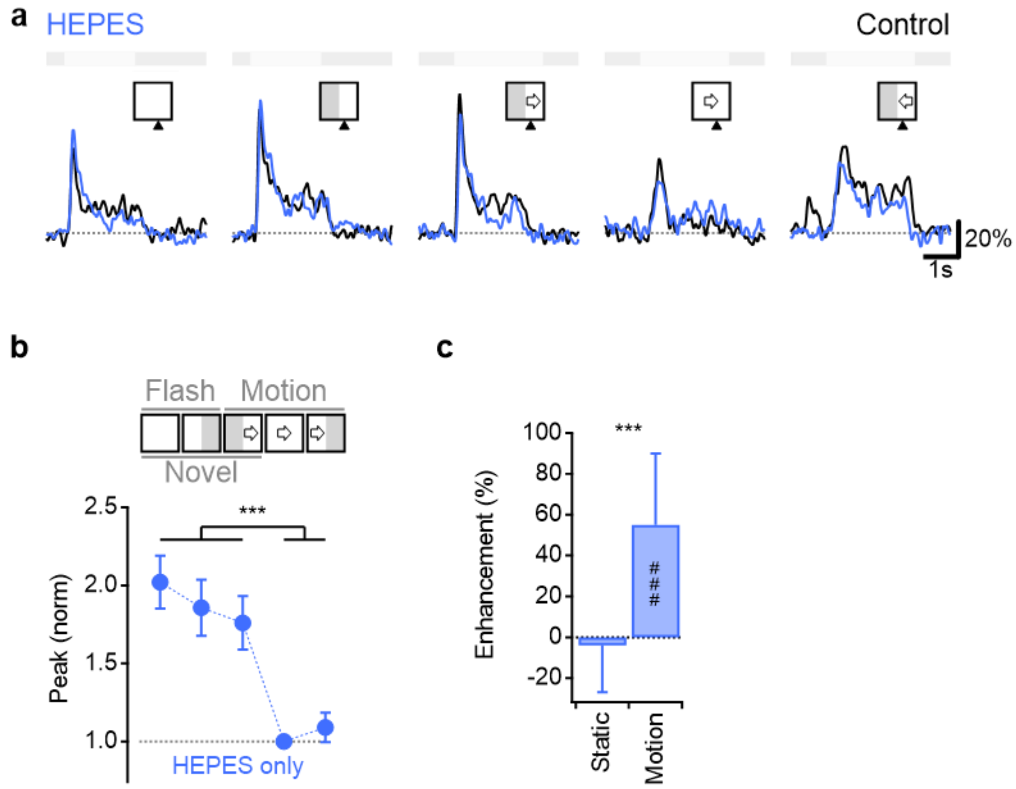


**Supplementary figure 6. HEPES alone does not eliminate edge effects. a** Representative glutamate responses before (black) and after (blue) perfusion with Ames medium containing 10 mM HEPES. **b** Peak glutamate signals (mean ± SEM), normalized by the peak amplitude of the full-field motion response in the presence of HEPES (one way ANOVA followed by Tukey test, *** FF flash vs. Edge $p=0.99$; FF flash vs. Emerging $p=0.15$; FF flash vs. Exit $p=3.6\times10^{-9}$; Edge vs. Emerging $p=0.22$; Edge vs. Exit $p=6.9\times10^{-9}$; Emerging vs. Exit $8.4\times10^{-6}$). **c** Enhancement (mean ± SD) of static edges and novel motion detection calculated from the peak of the responses with HEPES. ###$p=9.8\times10^{-6}$ change from 0%; ***$p=4.3\times10^{-5}$ difference between edge effects, paired t-test, n=12 ROIs from a male animal, age p233.

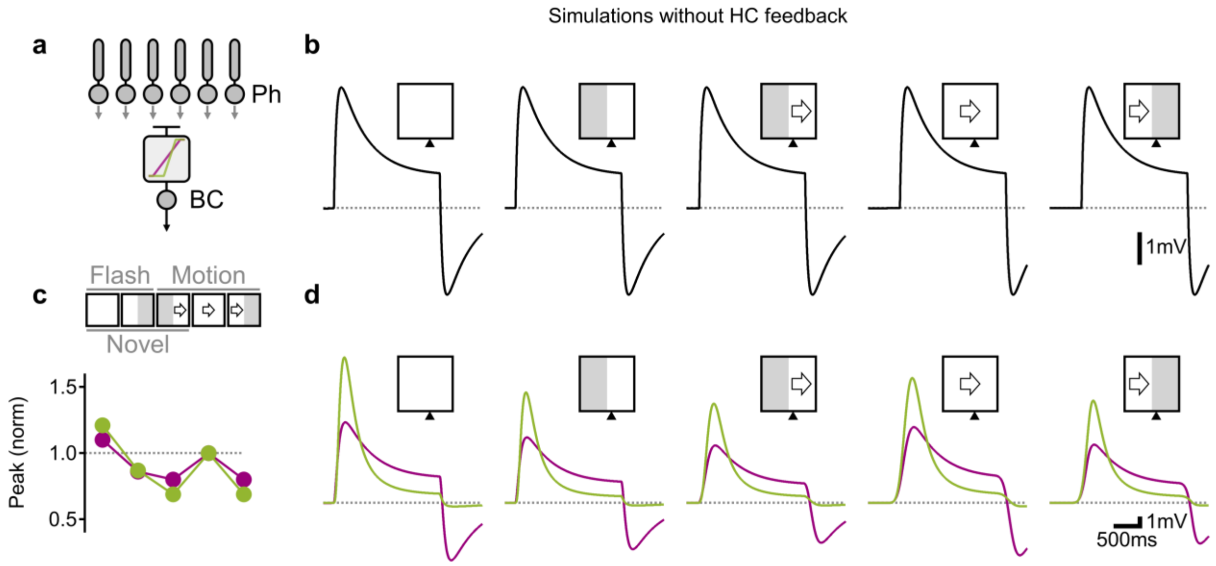


**Supplementary figure 7. Visual processing in a simulated retina that lacks horizontal cells replicates experimental findings of reduced edge effects. a** The simulated retinal circuit that did not include inhibitory components. Other network parameters and stimuli were left unchanged. **b** Responses from photoreceptors to static flashes and moving bars, in the presence or absence of a visual edge. **c** The peak depolarization, normalized by responses to the full-field moving bar for the transient (green) and sustained (red) BCs simulated as in **Fig. 4a-d**. **d** As in **b** for the two BCs. Horizontal cell surround was not absolutely required for generating smaller and slower responses to motion. Edge effects, however, were absent.

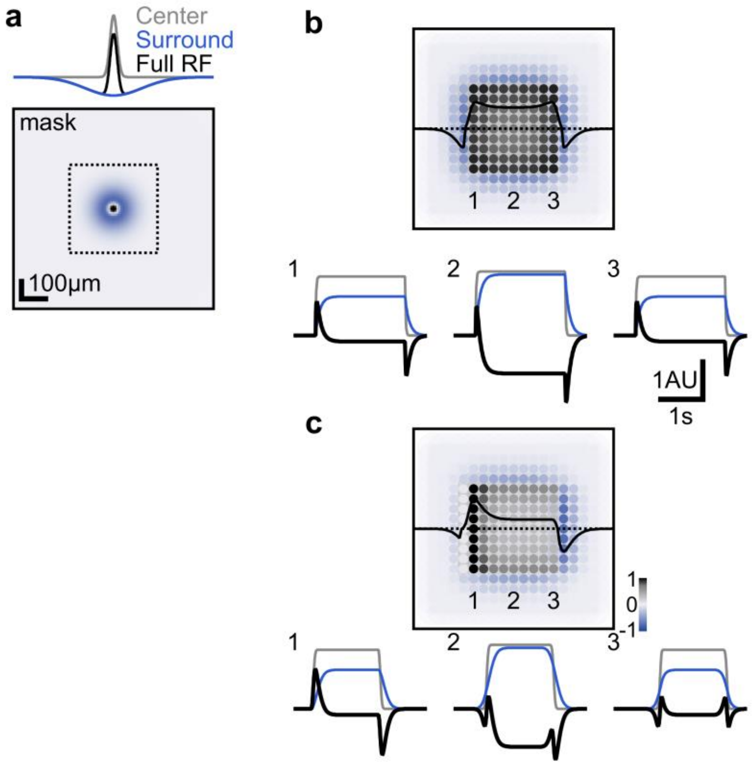


**Supplementary figure 8. Novel object enhancement is a general property of center-surround RFs. a** Example spatial extent of a simulated neuron with a linear center-surround RF organization. **b** Top, peak responses from a population of neurons with a similar RF structure to flashed stationary square (stimulus position marked in **a**). The black curve shows a horizontal activation profile of cells near the dotted line. Bottom, the temporal RF evolution in three example cells whose horizontal position is marked on top. Color coding as in **a**. **c** as in (**b**) for a moving bar.

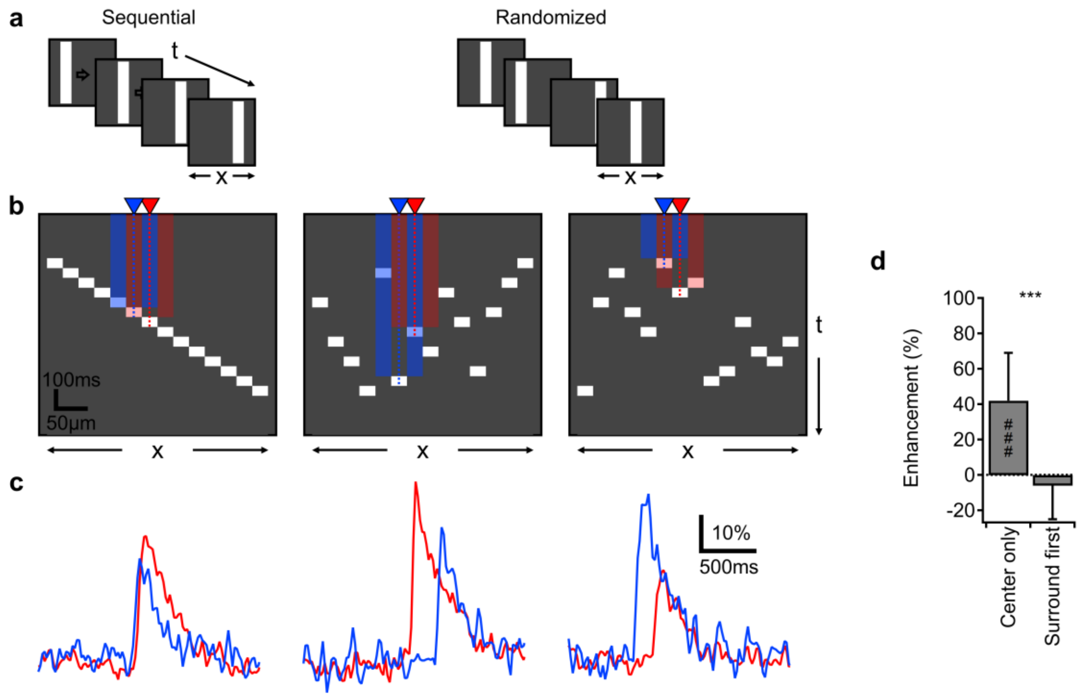


**Supplementary figure 9. Response amplitudes to a randomized presentation of a moving bar depend on the history of surround activation**

**a** Schematic of the stimulation paradigm. Each stimulation trial consisted of 14 25x350 µm bars flashed on the screen for 50 ms. The bars were shown sequentially to mimic a moving bar stimulus (left), or out of order (randomized, right). **b** Example spatiotemporal patterns for apparent motion (left) and two examples of shuffled sequences (middle and right). Superimposed are the horizontal positions of two neighboring ROIs. The position of their RF center is marked with arrows; dotted lines continue till the frame where the stimulus overlapped with RF center. The surround extends around the center, here illustrated with colored bars on adjacent spatial positions. **c** Mean responses from the ROIs and trials in **b**. Note that response amplitudes in shuffled trials were smaller when RF surround was activated prior to the center. **d** The ratio between response amplitudes in the shuffled presentation trials where the center was activated first ('Center only') or followed the surround ('Surround first') to the responses to apparent motion. Data are presented as mean values ± SD. ###$p=4\times10^{-14}$ change from 0%.***$p=1.7\times10^{-10}$ difference between center first/surround first trials (t-test, n=69 ROIs from 19 regions, 345 trials, 1 female animal, age p233).

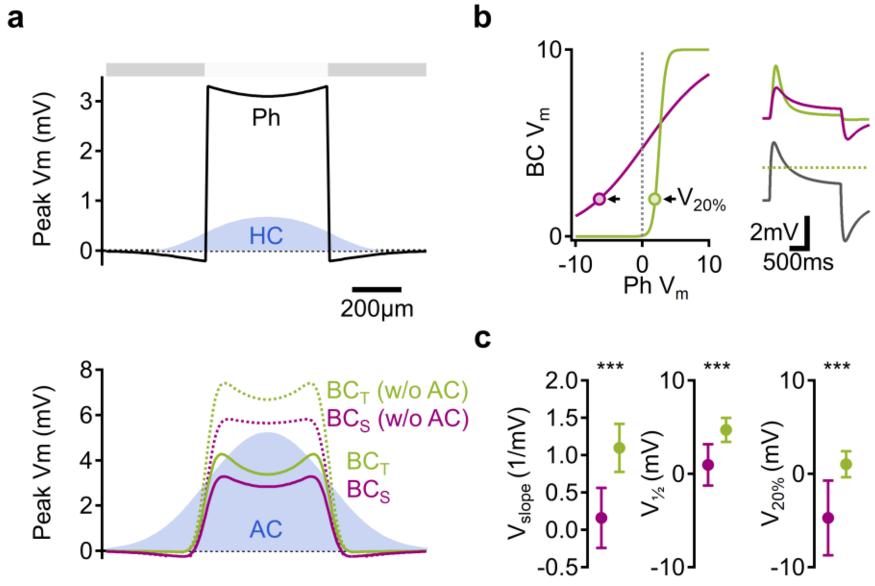


**Supplementary figure 10. Elevated affinity to neurotransmitter release promotes transient responses in simulated BCs. a** The artificial retinal circuit was activated with a spot (500µm in diameter). Top, peak photoreceptor and horizontal cell activation as a function of distance (polarity-inverted for presentation purposes). Bottom, BC (red-sustained, green-transient) activation vs. distance in models excluding (dotted) or including (solid) AC inhibition (blue). **b** Synaptic sensitivity (left) and the simulated light response (right) for the two BCs. V20% marks the photoreceptor voltage that depolarized the BC to 20% of the maximally attainable response (right, bottom, dotted). **c** The effects of the slope (left), half-peak (center) and V20% (right) of the Ph-BC input-output transformation on BC dynamics. ***two-tailed t-test, with Bonferroni's correction for multiple comparisons; $V_{slope}$: $p=1\times10^{-16}$, $V_{half}$:$p=2\times10^{-4}$, $V_{20\%}$: $p=7\times10^{-10}$. Data are presented as mean values ± SD. Transient BCs had a stronger and elevated threshold for activation.

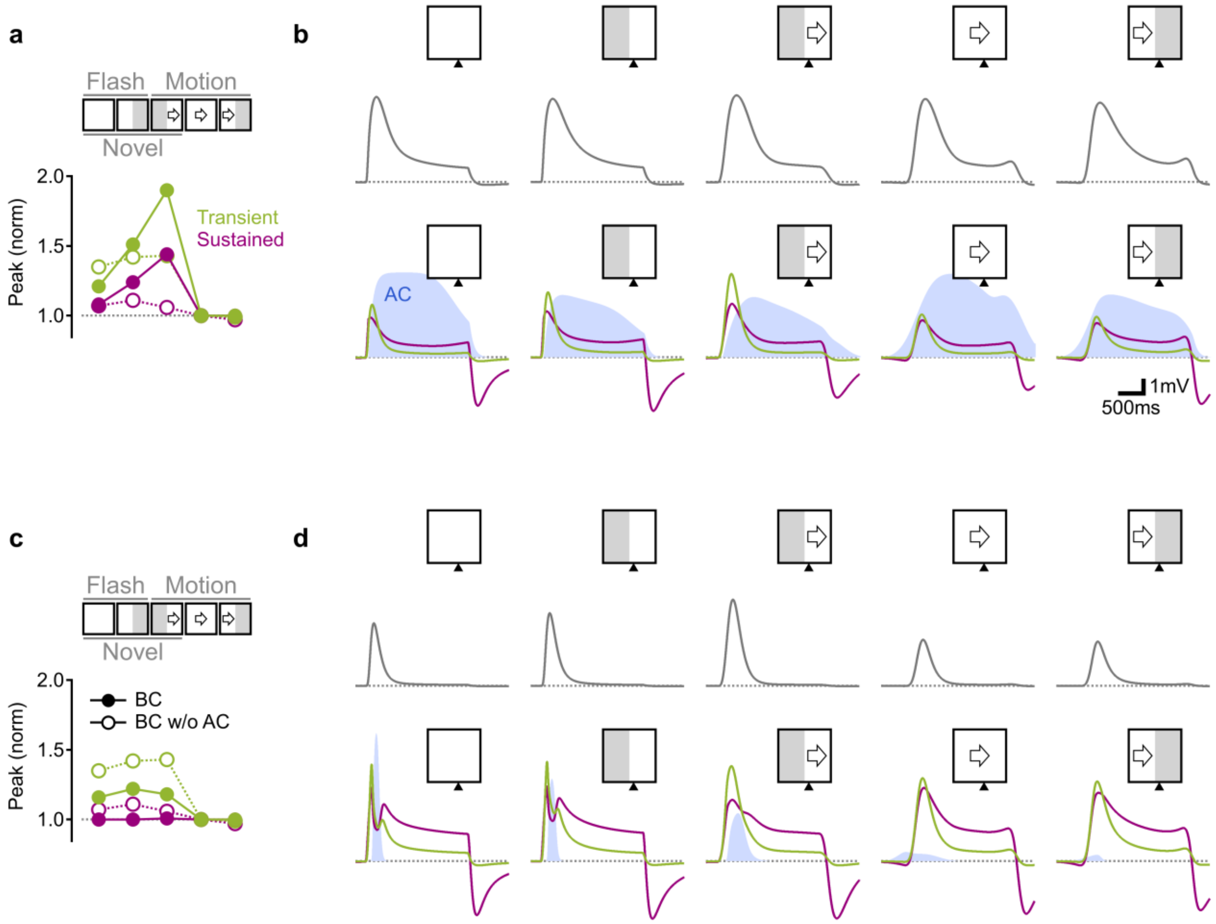


**Supplementary figure 11. Examples of the diversity of the simulated amacrine cell-mediated inhibition on signal processing in BCs. a-b** Simulated ACs with parameters that enhance the representation of emerging motion. In this model implementation, ACs had a fast rise time and were stimulated by sustained BCs (**b**, top), allowing them to have a pronounced inhibitory effect during responses to a full-field moving stimulus. **a** The peak depolarization, normalized by the response to the full-field moving bar for the transient (green) and sustained (red) BCs simulated as in **Fig. 4**. **b** Responses to static flashes and moving bars, in the presence or absence of a visual edge. Color coding as in **a**, AC input is shown in blue. **c-d**, as in **a-b** with ACs driven by a transient BC population (**d**, top). Here, the inhibitory arm had reduced drive during full-field motion and motion towards an edge.

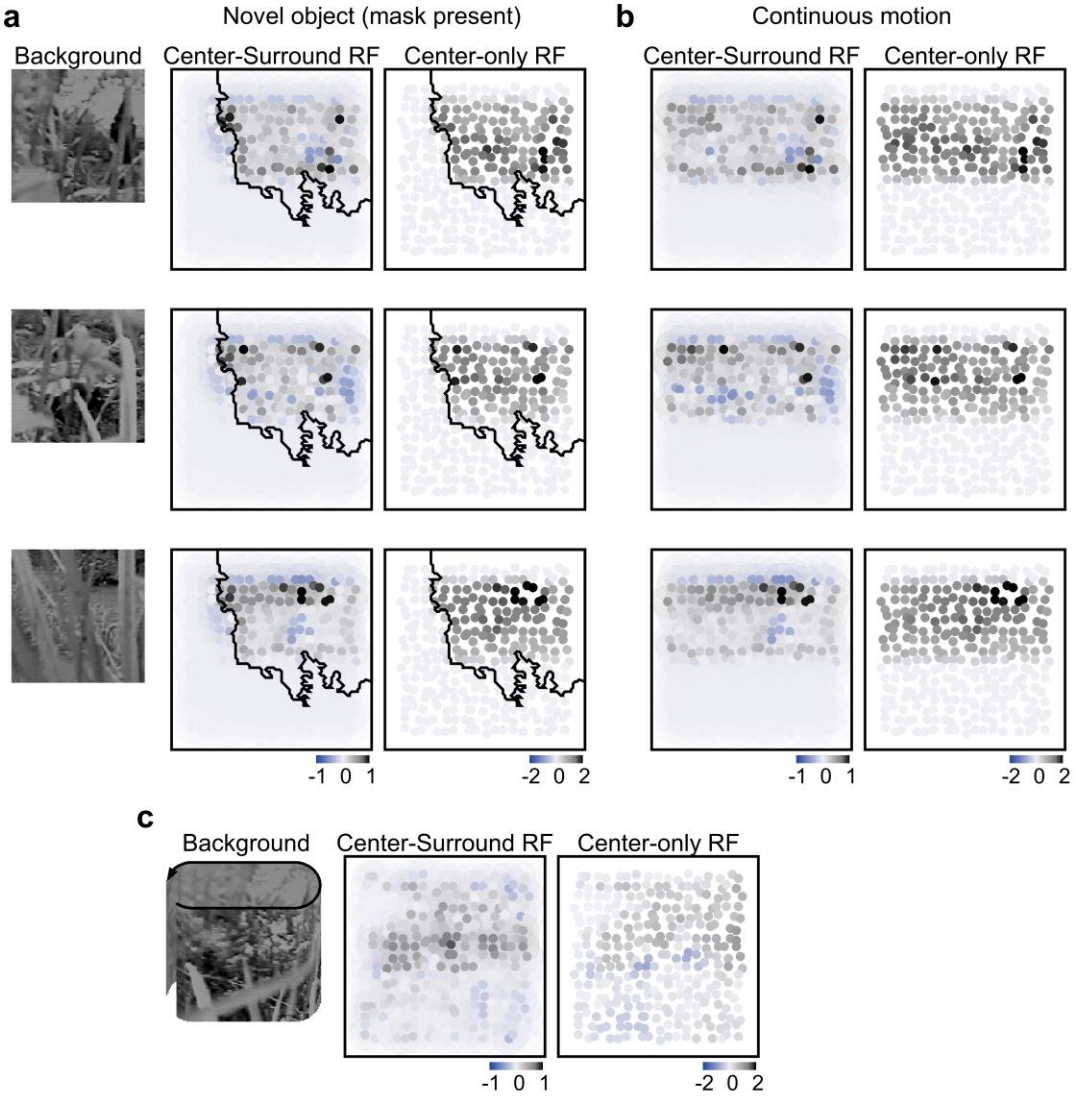


**Supplementary figure 12. Example responses of linear center-surround RFs to natural motion**

**a-b** Example population responses to natural movies with different backgrounds. The stimulus was the predator shown in **Fig. 5,** occluded by a mask (**a**) or moving unoccluded over the entire scene (**b**). **c** Responses to a global, horizontal translation of the background. No stimulus was shown.

| Cluster # | # ROIs near edges | Edge enhancement (**Fig. 2c**) | | | Emerging object enhancement (**Fig. 2d**) | | |
|---|---|---|---|---|---|---|---|
| | | Mean(±SEM) | raw p-value | p-values with Bonferroni's correction | Mean(±SEM) | raw p-value | p-values with Bonferroni's correction |
| **C1** | 6 | 0.13(±0.12) | 0.0144 | 0.2021 | 0.226(±0.2) | 0.0023 | 0.033571 |
| **C2** | 7 | -0.07(±0.09) | 0.1252 | >1 | -0.085(±0.09) | 0.3921 | >1 |
| **C3** | 32 | 0.014(±0.05) | 0.8764 | >1 | 0.18(±0.07) | 0.0032 | 0.0454 |
| **C4** | 27 | -0.087(±0.05) | 0.0083 | 0.1166 | -0.22(±0.06) | 0.0007 | 0.0106 |
| **C5** | 9 | -0.096(±0.1) | 0.0538 | 0.7545 | -0.369(±0.17) | $3.2\times10^{-5}$ | 0.00046 |
| **C6** | 12 | 0.064(±0.03) | 0.0662 | 0.9277 | 0.283(±0.13) | 0.0029 | 0.04197 |
| **C7** | 90 | 0.065(±0.03) | 0.0698 | 0.9777 | 0.377(±0.07) | $1.5\times10^{-8}$ | $2.1\times10^{-7}$ |
| **C8** | 70 | 0.061(±0.03) | 0.0713 | 0.9989 | 0.282(±0.05) | $3\times10^{-13}$ | $5.2\times10^{-12}$ |
| **C9** | 84 | 0.066(±0.02) | 0.0327 | 0.4588 | 0.196(±0.02) | $10^{-13}$ | $2.2\times10^{-12}$ |
| **C10** | 50 | 0.19(±0.03) | 0.0001 | 0.0026 | 0.211(±0.03) | $1.5\times10^{-8}$ | $2.1\times10^{-7}$ |
| **C11** | 51 | 0.07(±0.03) | 0.1223 | >1 | 0.119(±0.07) | 0.0017 | 0.02458 |
| **C12** | 42 | 0.059(±0.03) | 0.2034 | >1 | 0.008(±0.07) | 0.9064 | >1 |
| **C13** | 77 | 0.165(±0.02) | 0.0002 | 0.0039 | 0.374(±0.04) | $10^{-16}$ | $2\times10^{-15}$ |
| **C14** | 58 | 0.13(±0.04) | 0.0005 | 0.0079 | 0.339(±0.05) | $1.4\times10^{-9}$ | $1.9\times10^{-8}$ |

**Supplementary table 1**. Number of ROIs and p values (one way ANOVA followed by Tukey test) for the data presented in **Fig. 2c-d**.

| Condition (visual stimulus) | BC group | | p-values | | | | | | |
|---|---|---|---|---|---|---|---|---|---|
| | Transient | Sustained | Transient vs. sustained | Within transient BCs comparisons | | | Within sustained BCs comparisons | | |
| | | | | vs [2] | vs [3] | vs [4] | vs [2] | vs [3] | vs [4] |
| **Peak (Fig. 2e)** | | | | | | | | | |
| **FF flash [1]** | 1.472(±0.04) | 1.155(±0.02) | $<10^{-16}$ | 0.29 | $6\times10^{-10}$ | $<10^{-16}$ | 0.99 | 0.407 | $6\times10^{-4}$ |
| **Edge flash [2]** | 1.431(±0.03) | 1.186(±0.02) | $<10^{-16}$ | | $10^{-5}$ | $<10^{-16}$ | | 0.712 | $2\times10^{-4}$ |
| **Emergence [3]** | 1.282(±0.03) | 1.173(±0.02) | 0.017 | | | $<10^{-16}$ | | | $5\times10^{-6}$ |
| **Exit [4]** | 0.9658(±0.02) | 0.997(±0.01) | 0.97 | | | | | | |
| **Rise time (Fig. 2f)** | | | | | | | | | |
| **FF flash [1]** | 0.304(±0.02) | 0.306(±0.01) | 0.999 | 1 | 0.001 | $<10^{-16}$ | 0.964 | $8\times10^{-10}$ | $<10^{-16}$ |
| **Edge flash [2]** | 0.331(±0.01) | 0.342(±0.01) | 0.999 | | 0.001 | $<10^{-16}$ | | $2\times10^{-7}$ | $<10^{-16}$ |
| **Emergence [3]** | 0.44(±0.03) | 0.538(±0.02) | 0.219 | | | $<10^{-16}$ | | | $<10^{-16}$ |
| **Exit [4]** | 0.797(±0.04) | 0.798(±0.03) | 0.998 | | | | | | |
| **Decay time (Fig. 2f)** | | | | | | | | | |
| **FF flash [1]** | 0.456(±0.03) | 0.723(±0.03) | $2\times10^{-9}$ | 0.315 | 0.003 | $<10^{-16}$ | 0.996 | 0.986 | 0.988 |
| **Edge flash [2]** | 0.514(±0.04) | 0.718(±0.03) | 0.01 | | 0.776 | $2\times10^{-7}$ | | 1 | 0.67 |
| **Emergence [3]** | 0.633(±0.02) | 0.702(±0.02) | 0.541 | | | $5\times10^{-4}$ | | | 0.635 |
| **Exit [4]** | 0.76(±0.04) | 0.77(±0.03) | 0.999 | | | | | | |

**Supplementary table 2**. Statistics for the comparisons presented in **Fig. 2e-f**; p-values indicate the result of Tukey test. Numbers in brackets indicate different visual conditions.

| Condition (visual stimulus) | Control | GABA/Gly blockers | CNQX HEPES | Control vs. GABA/Gly | | Control vs. CNQX | | GABA/Gly vs. CNQX | |
|---|---|---|---|---|---|---|---|---|---|
| | Mean(±SEM) | | | raw p-value | p-values with Bonferroni's correction | raw p-value | p-values with Bonferroni's correction | raw p-value | p-values with Bonferroni's correction |
| **Peak (Fig. 5c)** | | | | | | | | | |
| **FF flash** | 1.35(±0.3) | 1.66(±0.2) | 1.4(±0.2) | 0.608 | >1 | 0.739 | 2.95 | 1 | >1 |
| **Edge flash** | 1.39(±0.3) | 1.46(±0.3) | 0.8(±0.1) | 0.11 | 0.44 | $3\times10^{-6}$ | $1.2\times10^{-5}$ | $2\times10^{-4}$ | $8\times10^{-4}$ |
| **Emergence** | 1.27(±0.3) | 1.34(±0.3) | 0.62(±0.1) | 0.105 | 0.4 | $10^{-11}$ | $4\times10^{-11}$ | $10^{-6}$ | $4\times10^{-6}$ |
| **Exit** | 0.97(±0.2) | 0.93(±0.3) | 0.62(±0.1) | 0.91 | >1 | $2\times10^{-5}$ | $8\times10^{-5}$ | 0.001 | 0.005 |
| **Rise time (Fig. 5d)** | | | | | | | | | |
| **FF flash** | 0.2(±0.1) | 0.25(±0.1) | 0.23(±0.2) | 0.92 | >1 | 0.857 | >1 | 0.994 | >1 |
| **Edge flash** | 0.32(±0.2) | 0.23(±0.2) | 0.27(±0.2) | 0.24 | 0.96 | 0.774 | >1 | 0.663 | >1 |
| **Emergence** | 0.3(±0.1) | 0.42(±0.1) | 0.52(±0.4) | 0.765 | >1 | 0.94 | >1 | 0.639 | >1 |
| **Exit** | 0.99(±0.1) | 0.95(±0.3) | 0.64(±0.3) | 0.181 | 0.724 | 0.001 | 0.005 | 0.01 | 0.04 |
| **Decay time (Fig. 5d)** | | | | | | | | | |
| **FF flash** | 0.46(±0.3) | 0.6(±0.1) | 0.5(±0.3) | 0.84 | >1 | 0.987 | >1 | 0.717 | >1 |
| **Edge flash** | 0.55(±0.4) | 0.51(±0.2) | 0.51(±0.3) | 0.466 | >1 | 0.173 | 0.692 | 0.904 | >1 |
| **Emergence** | 0.67(±0.1) | 0.62(±0.1) | 0.88(±0.4) | 0.965 | >1 | 0.047 | 0.188 | 0.192 | 0.768 |
| **Exit** | 0.92(±0.2) | 0.76(±0.1) | 0.73(±0.3) | 0.942 | >1 | 0.997 | >1 | 0.934 | >1 |

**Supplementary table 3**. Statistics for the comparisons presented in **Fig. 5c-d**; p-values indicate the result of ANOVA/Tukey tests performed on each visual condition individually.

| Pharmacology | Mean(±SD) | Vs. 0 | | Comparison with other pharmacological manipulations | |
|---|---|---|---|---|---|
| | | raw p-value | p-values with Bonferroni's correction | Vs. AC [2] | Vs. HC [3] |
| Edge enhancement | | | | | |
| Control [1] | 0.06(±0.2) | 0.954 | >1 | $8\times10^{-4}$ | $3\times10^{-12}$ |
| AC [2] | -0.13(±0.2) | $3\times10^{-4}$ | $9\times10^{-4}$ | | $2\times10^{-4}$ |
| HC [3] | -0.42(±0.2) | $<10^{-16}$ | $<10^{-16}$ | | |
| Emerging object enhancement | | | | | |
| Control [1] | 0.31(±0.2) | $<10^{-16}$ | $<10^{-16}$ | 0.576 | $10^{-4}$ |
| AC [2] | 0.42(±0.3) | $2\times10^{-7}$ | $7\times10^{-7}$ | | $5\times10^{-5}$ |
| HC [3] | 0.02(±0.2) | 0.182 | 0.548 | | |

**Supplementary table 4**. Statistics for the comparisons presented in **Fig. 5e**. Numbers in brackets indicate different phanrmacological conditions, corresponding p-values indicate the result of Tukey test.
