# Supplementary material for: Classical center-surround receptive fields facilitate novel object detection in retinal bipolar cells
Source: Nat Commun. 2022 Sep 26;13:5575. doi: 10.1038/s41467-022-32761-8 (PMC9512824; doi:10.1038/s41467-022-32761-8)
Supplement: Supplementary file 3 — Reporting Summary [file 41467_2022_32761_MOESM3_ESM.pdf]

nature portfolio

Corresponding author(s): Alon Poleg-Polsky

Last updated by author(s): Jun 24, 2022

Reporting Summary

Nature Portfolio wishes to improve the reproducibility of the work that we publish. This form provides structure for consistency and transparency in reporting. For further information on Nature Portfolio policies, see our Editorial Policies and the Editorial Policy Checklist.

Statistics

For all statistical analyses, confirm that the following items are present in the figure legend, table legend, main text, or Methods section.

n/a	Confirmed	
☐	☒	The exact sample size (*n*) for each experimental group/condition, given as a discrete number and unit of measurement
☐	☒	A statement on whether measurements were taken from distinct samples or whether the same sample was measured repeatedly
☐	☒	The statistical test(s) used AND whether they are one- or two-sided<br>*Only common tests should be described solely by name; describe more complex techniques in the Methods section.*
☒	☐	A description of all covariates tested
☐	☒	A description of any assumptions or corrections, such as tests of normality and adjustment for multiple comparisons
☐	☒	A full description of the statistical parameters including central tendency (e.g. means) or other basic estimates (e.g. regression coefficient) AND variation (e.g. standard deviation) or associated estimates of uncertainty (e.g. confidence intervals)
☐	☒	For null hypothesis testing, the test statistic (e.g. *F*, *t*, *r*) with confidence intervals, effect sizes, degrees of freedom and *P* value noted<br>*Give P values as exact values whenever suitable.*
☒	☐	For Bayesian analysis, information on the choice of priors and Markov chain Monte Carlo settings
☐	☒	For hierarchical and complex designs, identification of the appropriate level for tests and full reporting of outcomes
☐	☒	Estimates of effect sizes (e.g. Cohen's *d*, Pearson's *r*), indicating how they were calculated

Our web collection on statistics for biologists contains articles on many of the points above.

Software and code

Policy information about availability of computer code

Data collection	Igor Pro 8 (Wavemetrics)<br>Thorimage 4.1 (Thorlabs)
Data analysis	Igor Pro 8 (Wavemetrics)<br>R v3.6 (R foundation for statistical computing)

For manuscripts utilizing custom algorithms or software that are central to the research but not yet described in published literature, software must be made available to editors and reviewers. We strongly encourage code deposition in a community repository (e.g. GitHub). See the Nature Portfolio guidelines for submitting code & software for further information.

Data

Policy information about availability of data

All manuscripts must include a data availability statement. This statement should provide the following information, where applicable:

- Accession codes, unique identifiers, or web links for publicly available datasets
- A description of any restrictions on data availability
- For clinical datasets or third party data, please ensure that the statement adheres to our policy

The code for the visual stimulation and simulations is available on (https://github.com/PolegPolskyLab/) and on zenodo (https://zenodo.org/record/6814522). The full dataset was uploaded to zenodo (https://doi.org/10.5281/zenodo.6814536

Human research participants

Policy information about studies involving human research participants and Sex and Gender in Research.

Reporting on sex and gender	N/A
Population characteristics	N/A
Recruitment	N/A
Ethics oversight	N/A

Note that full information on the approval of the study protocol must also be provided in the manuscript.

Field-specific reporting

Please select the one below that is the best fit for your research. If you are not sure, read the appropriate sections before making your selection.

☒ Life sciences ☐ Behavioural & social sciences ☐ Ecological, evolutionary & environmental sciences

For a reference copy of the document with all sections, see nature.com/documents/nr-reporting-summary-flat.pdf

Life sciences study design

All studies must disclose on these points even when the disclosure is negative.

Sample size	Sample sizes for results presented on figures 1 and 2 were based on previous reports (Matsumoto et al 2019. Franke et al 2017). For figures 3 and 6, the computed sample size was 16, computed using Clara sample power analysis of a double-sided analysis assuming a p-value of 5% and a power of 80% to detect an effect of 50%. We assumed a standard deviation of 50%. Figures 4 and 5 are simulation figures and Figure 7 is a summary figure. We did not do a formal sample size analysis for supplementary figures.
Data exclusions	Fluorescence signals were averaged across repeated visual protocol presentations. Pixels with dF/F values <20% were exclude from analysis
Replication	No clear differences were observed across different mouse strains and sexes; the analysis was performed once before the initial submission on key data presented in figures 1 and 2. Animal surgery and tissue dissection was repeated by several investigators with similar outcomes; this was done in about 5 animals
Randomization	N/A - in the experiments where an effect of pharmacological manipulation was measured (figure 3), each animal was its own control. Data reported in other experiments was obtained from animals with similar genetic backgrounds (as we specified in each experiment), both sexes and from a similar age group
Blinding	In general, we did not blind the the researcher analyzing the data to the manipulations performed because of the nature of the experiments. In most experiments reported here, all recordings that meet the inclusion criteria were analysed; thus no blinding was possible. In a subset of the pharmacological manipulations performed in the study (Figure 3) the researcher analyzing the data examined the sequence of the pharmacological intervention after they performed the measurements. However, this was not an integral part of experimental design and the researcher had access to the experimental protocol. Because of these facts, the procedure doesn't meet the requirements of blinding. Therefore, no blinding was done in this study

Reporting for specific materials, systems and methods

We require information from authors about some types of materials, experimental systems and methods used in many studies. Here, indicate whether each material, system or method listed is relevant to your study. If you are not sure if a list item applies to your research, read the appropriate section before selecting a response.

Materials & experimental systems

n/a	Involved in the study	
☒	☐	Antibodies
☒	☐	Eukaryotic cell lines
☒	☐	Palaeontology and archaeology
☐	☒	Animals and other organisms
☒	☐	Clinical data
☒	☐	Dual use research of concern

Methods

n/a	Involved in the study	
☒	☐	ChIP-seq
☒	☐	Flow cytometry
☒	☐	MRI-based neuroimaging

Animals and other research organisms

Policy information about studies involving animals; ARRIVE guidelines recommended for reporting animal research, and Sex and Gender in Research

Laboratory animals	C57BL/6J https://www.jax.org/strain/000664 and Chat-Cre https://www.jax.org/strain/031661 of ages p21–120 of either sex
Wild animals	no wild animals were used in this study
Reporting on sex	Sex of each mouse used in this study was recorded; we did not observe an effect of sex on the principal findings.
Field-collected samples	No field collected samples were used in the study
Ethics oversight	All animal procedures were conducted in accordance with U.S. National Institutes of Health guidelines, as approved by the University of Colorado Institutional Animal Care and Use Committee (IACUC).

Note that full information on the approval of the study protocol must also be provided in the manuscript.
